# Finless Porpoise Bycatch and Stranding along the Shandong Peninsula, China, Based on Public Reports from 2000 to 2018

**DOI:** 10.3390/ani13243868

**Published:** 2023-12-15

**Authors:** Tao Zuo, Yongtao Li, Zhaolong Cheng, Jun Wang, Jianqiang Sun, Wei Yuan, Mingxiang Niu

**Affiliations:** 1 Yellow Sea Fisheries Research Institute, Chinese Academy of Fishery Sciences, Qingdao 266071, China; 2 Laboratory for Marine Ecology and Environmental Science, Pilot National Laboratory for Marine Science and Technology (Qingdao), Qingdao 266237, China

**Keywords:** finless porpoise, *Neophocaena asiaeorientalis sunameri*, stranding, bycatch, Shandong Peninsula

## Abstract

**Simple Summary:**

The East Asian finless porpoise is a common whale along Chinese coasts. The coast of the Shandong Peninsula is a substantial habitat for the species. However, there is relatively insufficient knowledge of and protection measures for the species population in this region. Based on public literature, media, and internet social websites, we collected and analyzed over two hundred reports on the stranding and accidental catching of finless porpoises along the coast of the Shandong Peninsula from 2000 to 2018. We found that bycatch and stranding incidents occurred widely across the peninsula throughout all seasons. The extensive use of fishing gear was the principal cause of porpoise mortalities and injuries along Shandong Peninsula. We recommend a comprehensive network consisting of an incident reporting system, fishing gear regulations, and scientific monitoring plans to protect the cetacean species in this region.

**Abstract:**

The Shandong Peninsula is located on the western coast of the Pacific and is adjacent to the Bohai Sea (BS) and the Yellow Sea (YS) to the east. The East Asian finless porpoise *Neophocaena asiaeorientalis sunameri*, a subspecies of the narrow-ridged finless porpoise *N. asiaeorientalis*, is the dominant cetacean resident along the Shandong Peninsula. However, there is insufficient monitoring data to determine the status of the cetacean species in this region. Based on the publicly available literature, media, and internet social website, this study investigated the spatial–temporal distribution of porpoise stranding and bycatch along the coast of the Shandong Peninsula. Data on over five hundred porpoises from two hundred reports between 2000 and 2018 were compiled and analyzed. Results showed that the bycatch and stranding of porpoises occurred widely across the peninsula throughout all months and increased rapidly between 2010 and 2017. The incidents were more frequent in the area where the BS and YS converged during the spring and early summer than in other seasons. The mean body length of bycaught porpoises was smaller than that of those found stranded. Fishing activities could be the principal cause of local finless porpoise incidents. However, limited data hindered a quantitative evaluation of the living conditions of finless porpoises in this area. Establishing a comprehensive monitoring system, which includes standardized reporting, rescue operations, and scientific research, is essential to finless porpoise protection along the Shandong Peninsula.

## 1. Introduction

The East Asian Finless Porpoise *Neophocaena asiaeorientalis sunameri* is a small-toothed cetacean with a wide distribution. It inhabits the coastal waters of the Taiwan Strait, the East China Sea, the Bohai Sea (BS), and the Yellow Sea (YS) in China, as well as in the coastal waters of Korea and Japan [1,2]. The species was believed to be closely related to the flagship Yangtze Finless Porpoise *N*. a. *asiaeorientalis* species in the Yangtze River, as two subspecies of the narrow-ridged finless porpoise *N. asiaeorientalis* [3]. However, recent phylogenetic and genomic studies have suggested that these two subspecies may be distinct and independent species [4]. The narrow-ridged finless porpoise plays a vital role in monitoring of the general health status of coastal ecosystems and maintaining ecological balance and biodiversity. However, the marine finless porpoise, like its subspecies in the Yangtze River [5], faces multiple endangering factors that put its existence at risk as a vulnerable species [6,7,8,9]. These factors include bycatch, vessel strikes, habitat loss, degradation, and food shortages. The population of this species has significantly declined on the west coast of Korea [10] and in the Inland Sea of Japan [11] over the past few decades. As a result, its conservation status was upgraded from Vulnerable (VU) to Endangered (EN) by the IUCN/SSC in 2017. In some local waters, it is at a high risk of becoming “Critically Endangered” (CR) [12].

The Shandong Peninsula is situated in northern China, on the western shore of the Pacific Ocean, and shares a border with the BS and YS in the east. It also faces the Korean Peninsula and the Japanese archipelago across a vast stretch of sea. It has over two hundred bays and ten estuaries of seagoing rivers including the Yellow River, the largest river in northern China. There are numerous traditional spawning, nursing, and feeding grounds for the different economically significant fish species [13] and whales [14,15,16,17] in these habitats.

*N. a. sunameri* is the predominant marine mammal in the coastal waters of the Shandong Peninsula [15]. However, anthropogenic activities, such as coastal engineering, petroleum exploitation, and aquaculture, have significantly increased the pressure on the finless porpoise in this area. The finless porpoise is inherently vulnerable to hazards due to its small size and coastal distribution [6]. Local fishermen have reported a decrease in sightings of the porpoise, suggesting a potential decline in its population in the area [18].

Monitoring such highly mobile and cryptic cetaceans in the wild is challenging and costly, especially over large-scale oceanic areas. Public reporting networks for the stranding and bycatch of aquatic mammals could be a supplemental method for cetacean ecological and preservation research. These networks could offer valuable scientific information on the status of cetaceans, especially for species with limited data [19,20,21,22]. Long-term reporting data have the same scientific reference value as at-sea scientific surveys, which could provide insights into cetacean diversity, distribution, and migration [19,20]. Similar reporting networks have been established in Korean waters [23], Hong Kong, Taiwan (http://tcsn.whale.org.tw accessed on 9 January 2019), and Hainan Island (http://www.cetacean.csdb.cn accessed on 9 January 2019) in the southern region of the China Seas [6,24,25,26]. However, the marine cetacean along the Shandong Peninsula is still data-deficient, except for a few studies on its species distribution in the 1970s–1980s [14,27,28,29] and in recent years [18,30,31]. They have not received the same level of conservation attention as their freshwater subspecies, the Yangtze finless porpoise. Regional information on finless porpoise bycatch and stranding is scattered in the media, literature, and other public platforms, covering various topics such as physiology and phylogenetics [32,33,34,35,36,37]. Therefore, constructing a reporting network is necessary for incidental events of standing and bycatch, which will be valuable for subsequent research and conservation efforts for the finless porpoise.

This study investigates stranding and bycatch incidents involving finless porpoises over the past twenty years along the Shandong Peninsula. Data from various sources, including the media, literature, and social websites, were analyzed to achieve two main objectives: Firstly, this study aims to understand the conservation status of finless porpoises regarding incidents of stranding, bycatch, and injury. The second aim is to establish an organized incident-reporting network for marine cetaceans and to provide insights for future decision making on the conservation and management of finless porpoises in the area.

## 2. Materials and Methods

### 2.1. Study Area

**Figure 1:**
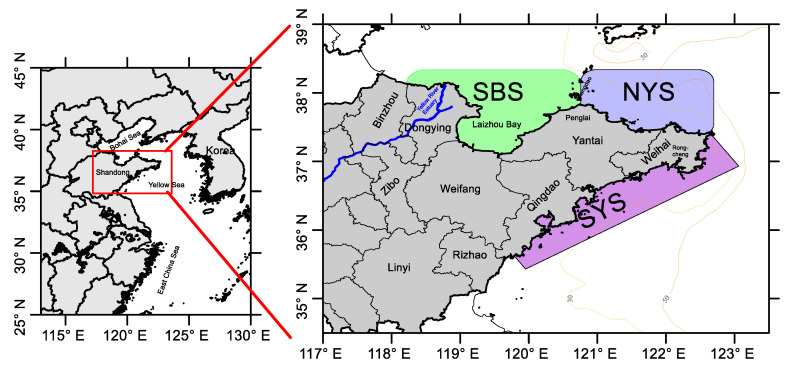
Map of the study area showing the regions analyzed.

The Shandong Peninsula is located on the western coast of the Pacific Ocean, within the coordinates of 119°16′–122°42.3′ E and 35°05′–37°50′ N (Figure 1). It has a coastline of approximately 3345 km and consists of seven coastal districts: Bingzhou, Dongying, Weifang, Yantai, Weihai, Qingdao, and Rizhao. The region is characterized by a consistent eastward coastal current in the BS, and a southward current in the YS, following 30 m isobaths along the peninsula [38]. During winter, the Bohai Strait brings northward saline YS Warm Water into the BS [39]. The region is known for its important fishing grounds, including the Laizhou Bay fishing ground, Yan(tai)-Wei(hai) fishing ground, and Wei(hai)-Qing(dao) fishing ground, due to the confluence of multiple water systems [13]. For statistical analysis, the coastal areas were divided into three geographical sections: SBS (Laizhou Bay and Yellow River Estuary in the southern part of the BS), NYS (the northern YS from Penglai to Rongcheng), and SYS (the southern YS from Rongcheng to Qingdao) for analysis and comparison. The study defined four seasons as follows: Spring (March to May), Summer (June to August), Autumn (September to November), and Winter (December to February).

### 2.2. Data Collection

In this study, stranding applied to an individual porpoise, whether alive or dead, who had been beached or washed up on the shore [25]. Bycatch applied to an individual, alive or dead, entangled in fishing gear [25]. Rescue referred to animals injured when discovered, but later successfully returned to the sea [25]. We collected extensive data from various sources, including the published literature, interviews with local fishermen, media reports, and social websites. The media consisted of newspapers, broadcasting, and television. The literature consisted of published articles in academic journals and dissertations. Social websites encompassed internet forums, blogs, and social platforms, as well as online reports on official websites.

Our Microsoft Access database only contained records verified by experts or confirmed with detailed descriptions, photos, or videos. Each record entry in the database included the porpoise number, sighting date, location of discovery, and physical characteristics, such as length, weight, and sex. We also recorded the life status of the porpoise (alive, dead, injured, or decomposed) and, if available, possible causes for injury or death. Missing information was registered as “undetermined” in the database. Events without the exact geographical coordinates were categorized based on the smallest relevant administrative unit. In cases where there were multiple reports of an event, we adopted the original reporting or discovering date. A mass event in this study referred to two or more porpoises caught or stranded at the same place and on the same date.

### 2.3. Data Analysis

In the following statistical analysis, we focused on data from 2000 to 2018. The locations of events were digitized on a map to visualize the spatial distribution of stranding and bycatch. The encounter rates of stranding and bycatch were calculated as the number of individuals per unit distance (ind./100 km) of the coastline [40]. The coastline length for each district was derived from the Shandong Administration of Surveying Mapping and Geo-Information.

The records of bycatch and stranding Incidents were categorized by year and month to identify annual and seasonal patterns. To explore the regional and seasonal differences in incident occurrence, an ANOVA with a statistical significance level of 0.05 was employed. Additionally, the body length distributions of stranding and bycatch were compared using the Kolmogorov–Smirnov test, with a statistical significance level of 0.05. The above analyses were conducted using STATISTICA 6.0 (StatSoft, Inc., Tulsa, OK, USA).

## 3. Results

### 3.1. Data Overview

Data on 606 finless porpoises were collected from 240 reports of target fishing, bycatch, and stranding between 1958 and 2018. Most of the information came from the published literature and the media, as shown in Table 1. The following analysis excluded the records of 66 targeted fishing porpoises before 1985 and two bycatches before 2000. 

Between 2000 and 2018, about 326 bycatch and 203 stranding porpoises were recorded. However, only 150 bycatch and 165 stranding events had the exact location and date descriptions. There were about 14 mass events, consisting of 8 strandings (involving 19 individuals) and 6 bycatch incidents (involving 42 individuals).

### 3.2. Annual and Seasonal Variations

**Figure 2:**
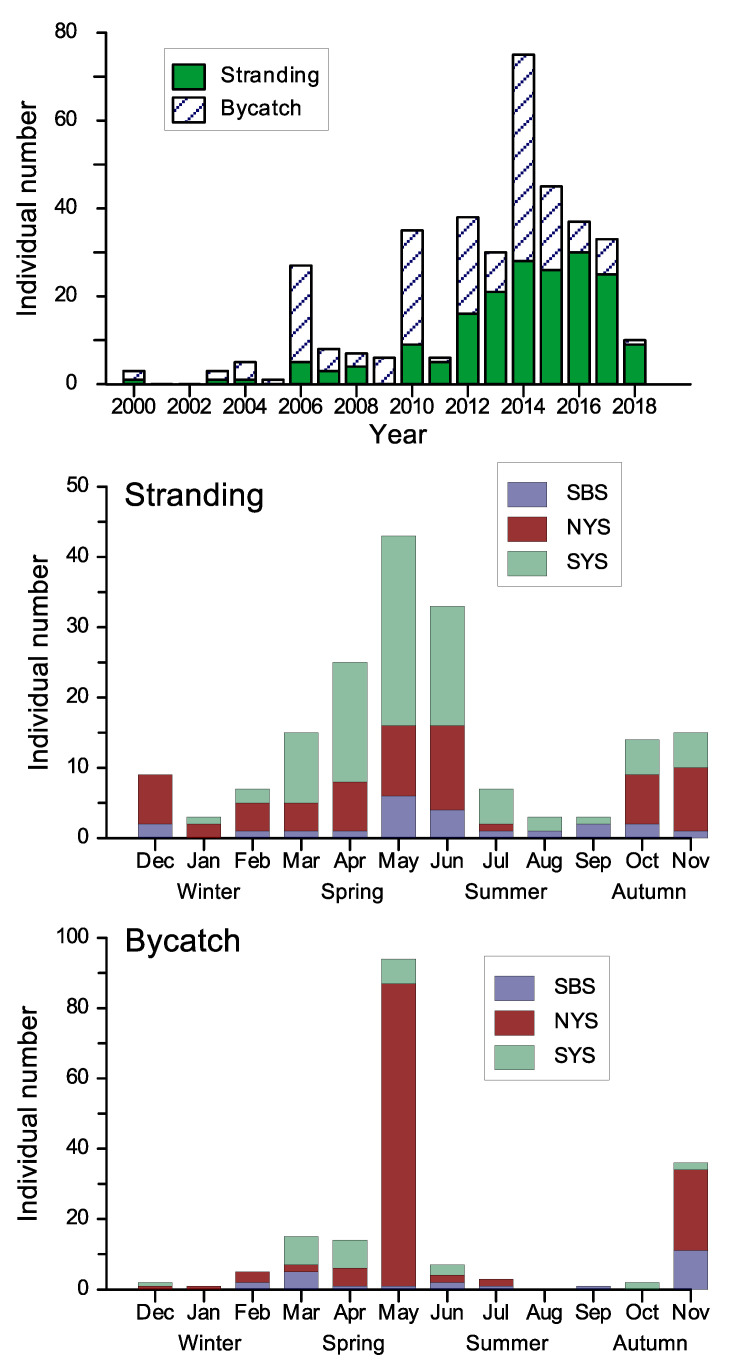
Annual and seasonal individual numbers of finless porpoise stranding and bycatch along the Shandong Peninsula.

About 184 stranding and 185 bycatch porpoise individuals had information on the reporting year available. Figure 2 illustrates a significant increase in porpoise incidents since 2010. On average, there have been approximately 30 finless porpoise incidents per year over the past eighteen years along the Shandong Peninsula.

**Figure 3:**
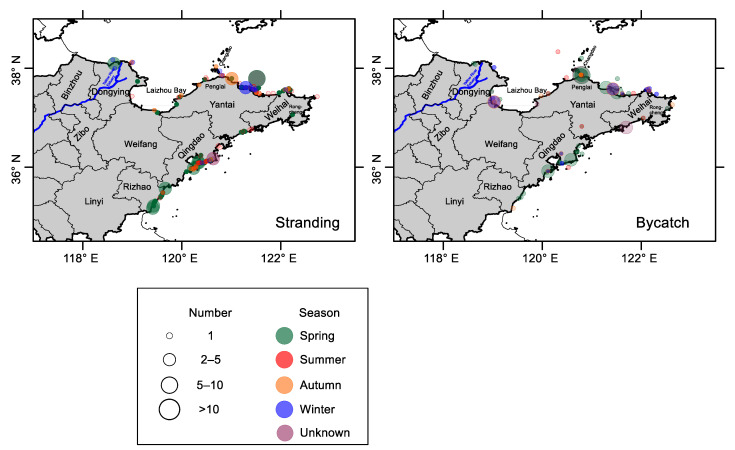
Distribution of finless porpoise stranding and bycatch from 2000 to 2018 along the Shandong Peninsula.

Out of these incidents, exact dates were available for 177 strandings and 180 bycaught individuals. Both bycatch and stranding events occurred throughout the year, with a peak in May during the spring season (Figure 2). The maximum value of stranding occurred during spring and early summer (from April to June), primarily concentrated in SYS. The primary peak of bycatch in May was contributed mainly by Yantai and Weihai in NYS (Figure 2 and Figure 3).

### 3.3. Geographic Distribution

Table 2 and Figure 3 show that stranding and bycatch occurred throughout the coasts of the Shandong Peninsula. NYS had the highest number of bycatch cases, while SYS had the highest stranding incidence. Both the bycatch and stranding encounter rates were the highest in NYS. Yantai had the most stranding and bycatch cases among the six districts. The highest stranding encounter rate occurred in Rizhao (25.1 ind. 100 km^−1^ shoreline), followed by Yantai and Qingdao. The highest bycatch encounter rate occurred in Yantai (17.9 ind. 100 km^−1^ shoreline), followed by Weihai and Weifang. However, there was no significant difference in either stranding or bycatch among three regions (the BS, NYS, and SYS) or six districts (ANOVA, *p* > 0.05).

### 3.4. Status and Disposal

**Figure 4:**
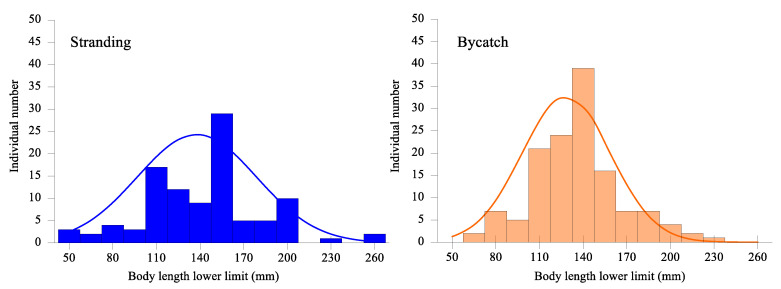
Body length-frequency distribution for stranded and bycatch finless porpoises between 2000 and 2018 along the Shandong Peninsula.

Out of the collected records, there were very few descriptions of the individual-level measurements of porpoises recorded (<5), apart from body length. In the study, the body-length records were available for 237 porpoises (102 strandings and 135 bycatches). Figure 4 shows that the body length of individual porpoises ranged from 50 cm to 260 cm. Over 85% of the recorded individuals had body lengths of between 100 cm and 200 cm. Additionally, over 63% of these individuals had body lengths of between 100 cm and 160 cm. The mean body length of stranded individuals was (137 ± 41 cm), which was significantly higher than that of bycatch individuals (127 ± 30 cm; Kolmogorov–Smirnov test, *p* < 0.001). Furthermore, there were significant differences in mean body length among the six districts (one-way analysis of variance, F = 2.45, df = 6, *p* = 0.025), with Qingdao having the highest value, followed by Rizhao, Dongying, Yantai, Weihai, and Weifang.

**Figure 5:**
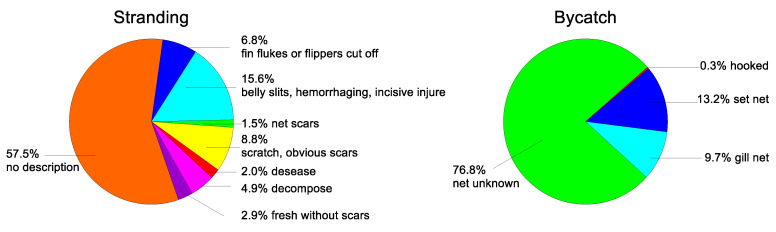
Mortality causes from the stranding and bycatch of finless porpoise along the Shandong Peninsula.

From the stranding records with a description of the cause of death, only four porpoises died of postpartum infection. Some stranding individuals had scars on their bodies, likely from fishing gear such as propellers, nets, or vessel collisions. Most of the bycatch porpoise’s bodies had visible net marks or physical injuries (Figure 5). The bycatch porpoises were reported to be injured mainly by net cages in aquatic waters or entangled in fishing nets while at sea.

A total of 83 strandings and 277 bycatch individuals had descriptions of their carcass’ disposal or rescue. Among them, only 66 individuals found were alive, and 42 were released (20 from stranding, 22 from bycatch). However, only ten individuals (four from stranding and the rest from bycatch) were in relatively good physical condition when released. The remaining dead individuals consisted of 59 strandings and 235 bycatches, of which approximately 74.8% were preserved or frozen in universities, aquaria, museums, and research institutes, 12.2% buried in situ, and 13% found for sale without authorization.

## 4. Discussion

### 4.1. Spatial and Temporal Variation

This study compiled the bycatch and stranding incidents of finless porpoises through an extensive review of public reports over the past two decades along the Shandong Peninsula. Despite some limitations in the data, they were still valuable for their insights into the temporal and spatial distribution of the porpoise population in the area. The results indicated that finless porpoises can be found year-round along the coasts of the Shandong Peninsula. Finless porpoise incidents occurred with relatively high frequency in the Laizhou Bay, Yan(tai)-Wei(hai), and Qing(dao)-Rong(cheng) coastal waters (Figure 2), which is consistent with previous studies in the 1980s [14,28]. These regions are known as highly productive fishing grounds [13] in zones where river runoff and oceanic waters are mixed between the YS and BS [38,39]. Moreover, they are also the substantial breeding areas for the finless porpoise [41].

As shown in Figure 2 and Figure 3, porpoise incidents were relatively higher during the spring and early summer than in other seasons. These seasonal variations in finless porpoise occurrences could be related to factors such as prey movements, freshwater discharge, or monsoon rains [42]. The porpoise’s prey, such as the fishes *Liza haematocheila*, *Sillago sihama*, and *Lateolabrax maculatus*, prefer to gather near the shore and reproduce at this time of the year [24]. Interestingly, a porpoise aggregation reported in June 2017 coincided with the spawning and schooling of *L. haematocheila* in Laoshan Bay, Qingdao. Additionally, there was a sub-peak in the bycatch and stranding of porpoises during the autumn season (Figure 2), which may be related to the migration of fish and continuous freshwater discharges. Historical records suggest that porpoises prefer to pursue highly migratory fish northward into the BS during early spring and southward into the NYS during fall and winter [14,15]. Schools of finless porpoises were observed swimming southeastward near Changdao Islands between the BS and NYS on 15 January 1999 [43]. Continuous freshwater discharges may be another factor promoting increases in porpoise levels. There were few cetacean records in local chronicles until the late 1800s, when the Yellow River emptied into the BS [17]. Local fishermen confirmed that finless porpoises almost disappeared near the Yellow River Estuary before implementing the Yellow River Water-Sediment Regulation project in 2002, especially during the severe drought period of 1987–2000.

### 4.2. Bycatch and Stranding 

**Figure 6:**
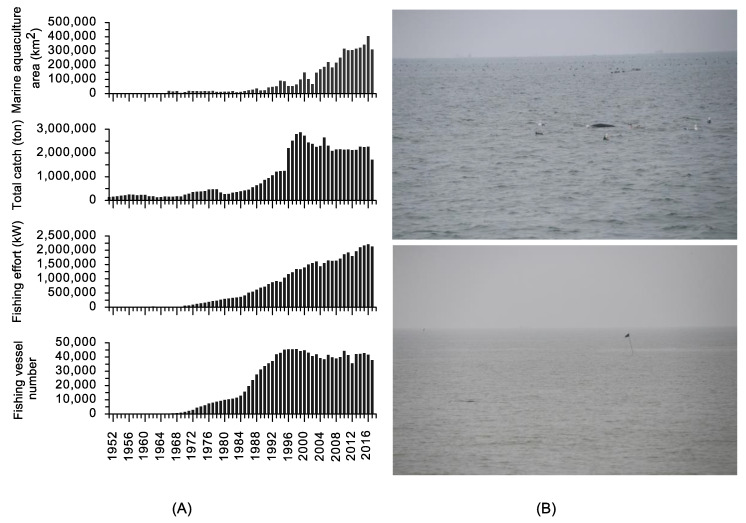
Annual trend of aquaculture area and fishing efforts along Shandong Peninsula (**A**) and a photo of a finless porpoise swimming close to nets in the aquaculture zone outside Rongcheng (**B**).

This study documented incidents of bycatch and stranding throughout the Shandong Peninsula (Figure 3). This continuous occurrence of incidents may be related to the extensive construction of aquaculture facilities and fishing enclosures (see Figure 6). Offshore aquaculture has been expanding rapidly along these coasts since the late 1990s, covering almost the entire traditional habitats of finless porpoises, from 10 m isobaths to 30 m isobaths. According to the China Fishery Statistics Yearbook of 2017, the total offshore aquaculture area in the Shandong Peninsula reached 3153 km^2^, with offshore and ordinary cages accounting for 1,970,068 m^3^ and 1,627,355 m^2^, respectively. Additionally, this region hosts over 38,410 marine fishing vessels with an annual fish catch of 1.74 million tons (Figure 6), representing one-fifth of China’s total marine catch. Such intensive aquaculture and fishing activities could lead to accidental entanglement and migration difficulties for finless porpoises, resulting in population declination and fragmentation of their distribution [7].

Fishery gear poses a significant threat to marine mammals worldwide, including the finless porpoise [21,22]. Evidence suggests that these porpoises experience incidental mortality directly or indirectly from fishing gear within their habitats [6,7,8,23]. Bycatch has resulted in an annual reduction in the porpoise population of over 15% in the Korean portion of YS [10]. In Ariake Sound and Tachibana Bay in Japan, gillnet entanglement alone has caused a population decline of 30–86% over three generations of porpoises [9]. Similarly, our results (Figure 5) indicate that the dominant causes of the porpoise mortalities and injuries along Shandong are interactions with gillnets, set nets, vessel propellers, and anti-predator facilities in aquaculture enclosures. In May 2014, about forty bycatch carcasses found at the Penglai port in Yantai showed evident scars from fishing gear [35]. However, the finless porpoise was the most commonly bycaught cetacean species along Chinese coasts, with the number of bycatch reaching more than 2000 individuals in 1994 [6]. This situation has not improved and may have worsened [24,25,26]. Due to the absence of a reliable regulatory and reporting network for the bycatch of marine cetaceans, the recorded number of porpoise incident events in this study is likely lower than the actual occurrence. In addition, similar to the situation in South Korea [44], most bycaught porpoises along the Shandong Peninsula were discarded at sea and rarely reported or taken back to the dock due to their low economic value and illegality, according to informal interviews with local fishermen. The individuals discarded or injured by collisions with vessels or propellers may drift to the beach via sea currents. This may explain the notable proportion of stranded individuals found with scars on their bodies (Figure 5).

Porpoise bycatch varied regionally and seasonally, as shown in Figure 2 and Figure 3. The spring peak of bycatch may be due to intensified fishing efforts and the presence of finless porpoises closer to the shore during this season [23,24]. Mass bycatch events may be related to the local use of set nets, bottom driftnets in the NYS and SYS, and gillnets in the BS, respectively. Moreover, entanglement in fishing gear may have a more negative impact on younger individuals [41]. Our study found that bycaught porpoises tended to be smaller in body length than those found stranded (Figure 4). Similar observations were reported for humpback dolphins in Australia [22] and South Africa [45], which suggests that younger dolphins may engage in more risky behavior compared to older individuals, and be more at risk of bycatch.

Cetacean stranding can result from various factors, including changes in habitat, the availability of prey, and illness or mortality from natural causes, as well as currents and winds [46]. However, limited information is available on the reasons for porpoise stranding along the Shandong coasts. Based on a few necropsy reports, stranded individuals have shown symptoms of illness, parasite infection, or difficult births [32,37], as well as injuries from fishing gear.

### 4.3. Conservation and Management

It is still challenging to assess the threat level to the finless porpoise population around the Shandong Peninsula, owing to the absence of long-term scientific monitoring data. However, some prior cases and conversations with fishermen [28,47] suggest that the current finless porpoise population along the Shandong Peninsula could be significantly lower than in the past. For instance, in June 1959, over forty porpoises were found aggregating in a small bay in Dongying during an ebb tide [28]. Questionnaire surveys with local fishermen indicate that the current porpoise population may be less than 20% of its size in the early 1980s [18]. Recent surveys estimated a finless porpoise density ranging from 0.044 to around 0.115 ind. km^−2^ in Laizhou Bay [30] and 0.169 ind. km^−2^ in the eastern Shandong Peninsula [31]. These densities were much lower than those estimated in western Korean waters [10] and Japanese waters [48,49,50,51]. Given the relatively low density of porpoise populations along the Shandong Peninsula and the increasing trend of incidents, conservation efforts for porpoises in this area are necessary.

Fortunately, China joined the International Whaling Commission (IWC) in the 1980s and terminated whaling immediately. Furthermore, the finless porpoise is on the list of second-class National Protected Animals in China, and there is growing interest in its conservation from the public, authorities, scientists, and wildlife protection organizations in Shandong. Increasing reports of stranding and bycatch in recent years (Figure 3) may be attributed partly to increased public attention. Moreover, some conservation and rescue measures have been implemented to mitigate current threats to porpoises. For example, an aquarium in Penglai has rescued and rehabilitated more than 20 individuals, and released six injured individuals successfully into the sea between 2012 and 2014 [52]. In addition, enhanced conservation regulations have reduced the illegal trade of porpoises, as evidenced by the first prosecution in June 2018 [53]. Furthermore, since 2018, fishing restrictions in the BS and YS have been extended from May to August, which aligns with the finless porpoise’s breeding season and the highest incidence of bycatch and stranding (Figure 3). These restrictions, by reducing fishing efforts, have proven effective in lessening fishery-related porpoise mortality in the Pearl River Estuary [54].

Based on this study, fishing activities pose a notable threat to the East Asian finless porpoise along the Shandong coastline. Since fishing efforts cannot be reduced significantly, it is essential to establish a local incident network for cetacean conservation. We recommend the following measures: (i) Implementing a routine and standardized reporting program under authorized laws and regulations. Improving spatial and temporal coverage of reported data, especially in traditional porpoise habitats and hotspots where incidents occur. (ii) Strengthening and integrating various stakeholders, including local fishery authorities, market regulations, aquariums, fishermen, trained volunteers, and mammal experts. (iii) Implementing local modifications and restrictions on fishery gear, such as installing mammal escape devices and acoustic deterrents to reduce unintentional entanglement. Our study identified specific fishing gear responsible for porpoise incidents, allowing for targeted modifications and restrictions based on the actual situation. (iv) Utilizing the data collected by the network and scientific monitoring projects to assess the population status of cetaceans and the effectiveness of these measures, which will facilitate improvements to the existing marine cetacean management and conservation system.

## 5. Conclusions

During the past two decades, public reports have indicated that finless porpoises could be found throughout the Shandong Peninsula. These porpoises have been stranded or bycaught unintentionally in fishing gear in all seasons and regions, with a higher occurrence in spring and early summer. The primary causes of accidental deaths and injuries of these porpoises are believed to be fishery facilities and activities. Smaller individuals are particularly affected by bycatch, which has a severe negative impact on them. Although there may be some bias in data from public reports, they still provide valuable insights into the current status of the data-limited finless porpoise along the Shandong Peninsula. Therefore, it is desirable to establish a comprehensive reporting and scientific monitoring network to protect this cetacean species.

## Figures and Tables

**Table 1:** Collection of incident events with individual number of finless porpoises along Shandong Peninsula.

	Media		Literature		Social Websites	
	Case Number	Individual Number	Case Number	Individual Number	Case Number	Individual Number
Stranding	111	123	4	33	48	47
Bycatch	37	61	12	237	16	28
Target-fished	0	0	7	66	0	0
Witnessed at sea	1	3	0	0	4	8

**Table 2:** Finless porpoise number and encounter rate (individuals/100 km shoreline) of stranding and bycatch along Shandong Peninsula between 2000 and 2018.

		Stranding		Bycatch	
		Number	Encounter Rate	Number	Encounter Rate
District	Binzhou	0	-	0	-
	Dongying	10	2.4	15	3.6
	Weifang	2	1.4	12	8.6
	Yantai	70	7.6	151	16.6
	Weihai	34	3.0	124	10.9
	Qingdao	59	6.8	20	2.3
	Rizhao	26	26.1	4	4.0
Defined region	SBS	22	2.1	49	4.7
	NYS	83	10.6	216	26.9
	SYS	96	6.1	61	4.0

## Data Availability

The data presented in this study are available on request from the corresponding author.
